# Mechanical ventilation interacts with endotoxemia to induce extrapulmonary organ dysfunction

**DOI:** 10.1186/cc5050

**Published:** 2006-09-22

**Authors:** D Shane O'Mahony, W Conrad Liles, William A Altemeier, Shireesha Dhanireddy, Charles W Frevert, Denny Liggitt, Thomas R Martin, Gustavo Matute-Bello

**Affiliations:** 1 Division of Pulmonary and Critical Care Medicine, University of Washington School of Medicine, Seattle, WA 98195; 2 Medical Research Service, VA Puget Sound Health Care System, 1660 S. Columbian Way, Seattle, WA 98108; 3 Division of Allergy and Infectious Diseases, University of Washington School of Medicine, Seattle, WA 98195; 4 Department of Comparative Medicine, University of Washington School of Medicine, Seattle WA 9815

## Abstract

**Introduction:**

Multiple organ dysfunction syndrome (MODS) is a common complication of sepsis in mechanically ventilated patients with acute respiratory distress syndrome, but the links between mechanical ventilation and MODS are unclear. Our goal was to determine whether a minimally injurious mechanical ventilation strategy synergizes with low-dose endotoxemia to induce the activation of pro-inflammatory pathways in the lungs and in the systemic circulation, resulting in distal organ dysfunction and/or injury.

**Methods:**

We administered intraperitoneal *Escherichia coli *lipopolysaccharide (LPS; 1 μg/g) to C57BL/6 mice, and 14 hours later subjected the mice to 6 hours of mechanical ventilation with tidal volumes of 10 ml/kg (LPS + MV). Comparison groups received ventilation but no LPS (MV), LPS but no ventilation (LPS), or neither LPS nor ventilation (phosphate-buffered saline; PBS).

**Results:**

Myeloperoxidase activity and the concentrations of the chemokines macrophage inflammatory protein-2 (MIP-2) and KC were significantly increased in the lungs of mice in the LPS + MV group, in comparison with mice in the PBS group. Interestingly, permeability changes across the alveolar epithelium and histological changes suggestive of lung injury were minimal in mice in the LPS + MV group. However, despite the minimal lung injury, the combination of mechanical ventilation and LPS resulted in chemical and histological evidence of liver and kidney injury, and this was associated with increases in the plasma concentrations of KC, MIP-2, IL-6, and TNF-α.

**Conclusion:**

Non-injurious mechanical ventilation strategies interact with endotoxemia in mice to enhance pro-inflammatory mechanisms in the lungs and promote extra-pulmonary end-organ injury, even in the absence of demonstrable acute lung injury.

## Introduction

Multiple organ dysfunction syndrome (MODS) is a leading cause of death among patients with sepsis [1,2]. MODS develops in critically ill patients, primarily in the setting of systemic insults, including sepsis, burns, pancreatitis, cardiopulmonary bypass, or acute respiratory distress syndrome (ARDS) [2-5]. MODS has been defined as progressive but reversible dysfunction of at least two organs that arises from an acute disruption of normal homeostasis, requiring intervention [1]. Not all patients with sepsis develop MODS, but the development of MODS increases the mortality of patients with sepsis [6]. The mechanisms that link sepsis and ARDS to the development of MODS are not well understood.

Recent studies suggest a possible link between mechanical ventilation and the development of MODS [7]. Imai and colleagues [7] demonstrated that rabbits develop renal and hepatic injury when subjected to intratracheal aspiration of hydrochloric acid followed by 8 hours of mechanical ventilation with tidal volumes of 15 to 17 ml/kg. This was associated with pulmonary and systemic increases in pro-inflammatory cytokines, such as monocyte chemotactic protein-1 (MCP-1), IL-8 and GRO, and evidence of apoptosis in the kidneys. This was the first study to show a link between mechanical ventilation strategies and systemic organ injury in animals, suggesting that mechanical ventilation at tidal volumes greater than those commonly used to treat patients with ARDS might contribute to both pulmonary and distal organ injury [8,9].

A separate line of research has recently shown that activation of innate immunity by bacterial products such as lipopolysaccharide (LPS) enhances the deleterious effects of mechanical ventilation in the lungs of mice and rabbits [10-12]. Rabbits treated with intravenous LPS show enhanced lung injury in response to mechanical ventilation using tidal volumes of 10 to 15 ml/kg. This increase in lung injury is associated with activation of the nuclear transcription factors NF-κB and AP-1 in the lungs [10,11]. In mice, a synergism between intratracheal LPS and mechanical ventilation is seen even with tidal volumes of 10 ml/kg, which are similar to those used in humans without ARDS [12]. Thus, mechanical ventilation synergizes with systemic and intratracheal LPS in the induction of acute lung injury.

Mechanical ventilation is emerging as a factor that can have systemic consequences, such as distal organ injury, in addition to its ability to enhance local injury induced by bacterial products in the lungs. An important question is whether mechanical ventilation at tidal volumes similar to those used in humans without ARDS synergizes with circulating LPS in the development of distal organ dysfunction. This possibility is clinically important because bacteremia and/or circulating bacterial products, such as LPS, are present in the circulation of critically ill humans [13-16]. Many critically ill patients with sepsis who have not yet developed ALI or ARDS are ventilated with tidal volumes of 10 ml/kg. Our studies demonstrating synergism between mechanical ventilation and endotoxemia and the studies by Imai and colleagues demonstrating a link between mechanical ventilation and MODS raise the possibility that these patients may be at risk for developing MODS.

The goal of the present study was to determine whether the combination of a low dose of systemic LPS, which does not cause lung injury by itself, with a minimally injurious mechanical ventilation strategy, would result in the development of lung injury or distal organ dysfunction. We used a mouse model of mechanical ventilation to simulate critically ill patients with sepsis who do not meet criteria for lung protective ventilation and who are being ventilated with tidal volumes of 10 ml/kg. We used this mouse model to determine whether mechanical ventilation at low tidal volume alone or in the presence of low-dose endotoxemia is associated with distal organ injury.

## Materials and methods

### Animal protocol

All the animal protocols were approved by the Animal Care Committee of University of Washington and the VA Puget Sound Healthcare System. Male C57BL/6 mice weighing 25 to 30 g received intraperitoneal injections of either PBS or 1 μg/g of *E. coli *LPS, O111:B6 (Sigma Chemical Co, St Louis, MO, USA). Immediately afterwards, the mice were treated with 1 ml subcutaneous of lactated Ringer's solution for fluid replacement. The mice were returned to their cages with free access to water and food. After 14 hours, the mice were anesthetized with inhaled isoflurane. The larynx was revealed and the trachea was intubated orally with an 18-gauge Vialon^® ^angiocath (BD, Franklin Lakes, NJ, USA). Placement of the catheter in the trachea was verified by detecting the movement of a 100 μl bubble of water located inside a syringe connected to the catheter, and by measurement of the mixed expired CO_2 _(MECO_2_) with a capnograph (Novametrics Medical Systems Inc, Wallingford, CT, USA). Once intratracheal intubation had been confirmed, the animal was mechanically ventilated with a rodent ventilator Type 845 (Mini-Vent, Cambridge, MA, USA) with the following settings: tidal volume, 10 ml/kg; respiratory rate, 150 breaths/minute; fraction of inspired oxygen, 0.21; and positive end-expiratory pressure, 0. Airway pressures, rectal temperature, and MECO_2 _were monitored continuously. In preliminary studies this ventilation strategy produced normal arterial blood pH values (7.36 ± 0.08, *n *= 4). The respiratory rate was adjusted to maintain the MECO_2 _between 10 and 15 Torr. The body temperature was maintained between 37 and 38°C with external heating. At one hour after the onset of mechanical ventilation, the mice received an initial subcutaneous fluid bolus of 0.15 ml of a 1:1 mixture of 5% dextrose and lactated Ringer's. Additional subcutaneous boluses of 0.15 ml were administered every 30 minutes. The mice were ventilated for six hours, and then killed with pentobarbital (120 mg/kg intraperitoneally). The mice were exsanguinated by direct cardiac puncture, the thorax was opened, and the left lung was removed and placed in 1 ml of protease inhibitor solution (Complete™; Roche Applied Science, Indianapolis, IN, USA). The right lung was lavaged with PBS, removed from the thorax, suspended from the fixation apparatus, and fixed with 4% paraformaldehyde at a constant pressure of 15 cmH_2_O [17]. The abdomen was incised, and one lobe of the liver and the right kidney were removed. The capsule of the kidney was pierced several times and the tissues were placed in 4% paraformaldehyde.

### Experimental design

The mice received intraperitoneal LPS (1 μg/g), or PBS as described above. After 14 hours they were either allowed to breathe spontaneously or mechanically ventilated for six hours. The experimental design included four groups: PBS followed by spontaneous breathing (PBS), PBS followed by mechanical ventilation (MV), LPS followed by spontaneous breathing (LPS), and LPS followed by mechanical ventilation (LPS + MV).

### Sample processing

The protocols used to process the bronchoalveolar lavage fluid (BALF) have been described [18]. The blood was spun at 1,000 *g*, and the plasma was stored in aliquots for determinations of cytokines and markers for hepatic and renal dysfunction. The left lung was homogenized for 60 s with a hand-held homogenizer. The homogenate was divided into two aliquots. One aliquot was vigorously mixed with a buffer containing 0.5% Triton X-100, 150 mM NaCl, 15 mM Tris-HCl, 1 mM CaCl_2_, and 1 mM MgCl, pH 7.40, incubated for 30 minutes at 4°C, and then spun at 10,000 *g *for 20 minutes. The supernatants were aliquoted and stored at -80°C for later cytokine measurements. The second aliquot was vigorously mixed with 50 mM potassium phosphate, pH 6.0, with 5% hexadecyltrimethyl ammonium bromide (Sigma) and 5 mM EDTA in water. The mixture was sonicated and spun at 12,000 *g *for 15 min at 25°C, and the supernatants were aliquoted and stored at -80°C for later myeloperoxidase (MPO) measurements.

### Measurements

Total BALF cell counts were performed with a hemocytometer, and differential cell counts were performed on cytospin preparations. Lung homogenate MPO activity was measured with the Amplex Red fluorimetric assay, in accordance with instructions from the manufacturer (Molecular Probes, Eugene, OR, USA). The total protein concentration in BALF was measured with the bicinchoninic acid method (BCA assay; Pierce Co., Rockford, IL, USA). IgM concentrations in BALF were measured with specific mouse immunoassays (R&D Systems, Minneapolis, MN, USA). The cytokines KC, macrophage inflammatory protein-2 (MIP-2), IL-6, IL-1β, and TNF-α were measured in lung homogenates and plasma with commercially available microspheres for a multiplex fluorescent bead assay (Luminex, Austin, TX, USA). The soluble Fas ligand (FasL) concentration in lung homogenates and plasma was determined with a specific murine FasL immunoassay (R&D Systems). Creatinine, alanine aminotransferase (ALT), and aspartate aminotransferase (AST) were measured in plasma samples at the clinical laboratory of the University of Washington with standard techniques.

Whole and cleaved caspase-3 were detected in lung homogenated by Western blotting, using polyclonal antibodies for cleaved caspase-3 and uncleaved caspase-3 (Cell Signaling Technology, Beverly, MA, USA). Immunohistochemistry for cleaved caspase-3 was performed with the Vector 'Elite' ABC-HP kit (Vector, Burlingame, CA, USA) using a murine-specific rabbit anti-active capase-3 (BD Pharmingen, San Jose, CA, USA) for detection, and goat anti-rabbit biotinylated antibody (Vector) for labeling, as described previously [17].

### Statistical analysis

The data are expressed as means ± SEM from at least three independent experiments. The data were analyzed by one-way analysis of variance followed by Fisher's protected least significant difference. *p *< 0.05 was considered significant.

## Results

All of the mice in the PBS (*n *= 5), MV (*n *= 6) and LPS (*n *= 5) groups survived for the duration of the experiments. In the LPS + MV group, two out of six mice died, both of them during the third hour of ventilation. The data below were generated from the surviving mice.

### Physiological response to mechanical ventilation

**Figure 1:**
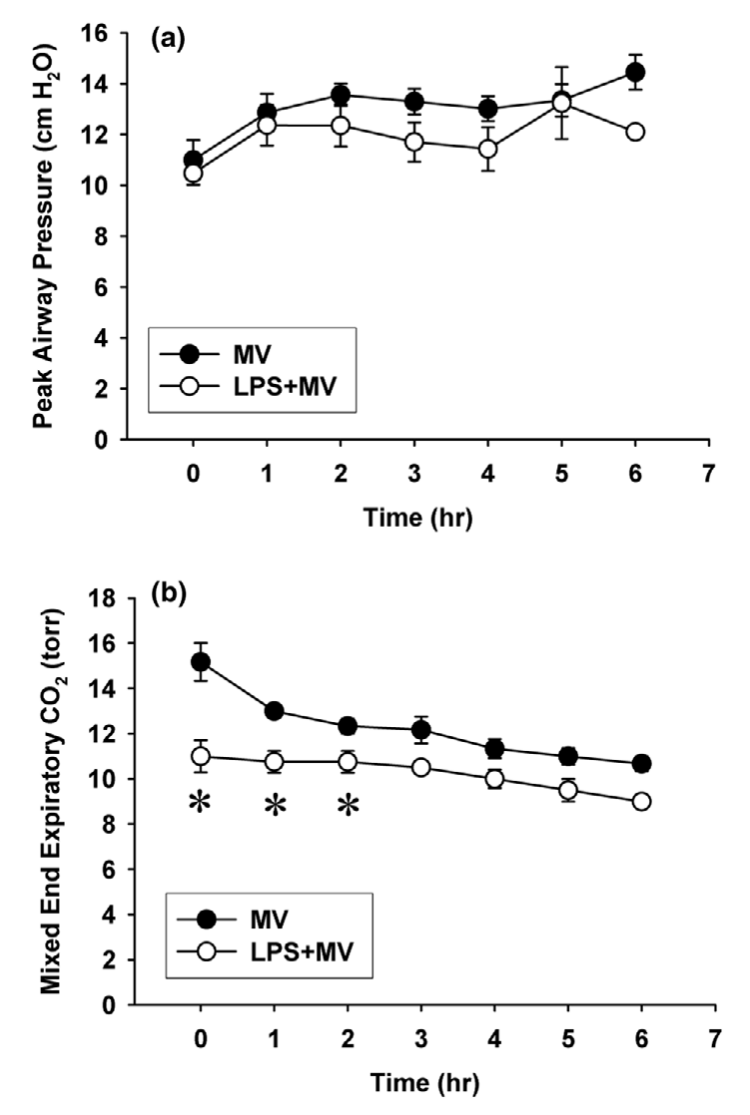
Physiological response to mechanical ventilation. Peak airway pressures **(a) **and mixed end-expiratory CO_2 _**(b) **in mice treated with intraperitoneal PBS followed 14 hours later by 6 hours of mechanical ventilation (MV), and in mice treated with intraperitoneal lipopolysaccharide (LPS; 1 μg/kg) followed 14 hours later by 6 hours of mechanical ventilation (LPS + MV). The tidal volume was 10 ml/kg and the fraction of inspired oxygen was 0.21. *p < 0.05 compared with the MV group.

In the ventilated groups (MV and LPS + MV), peak airway pressures were similar for the duration of the experiments (Figure 1a). At the beginning of the ventilation period, the mixed end-expiratory CO_2 _was significantly lower in the LPS + MV mice than in the MV mice (*p *< 0.05) and remained lower for the duration of the experiment (Figure 1b).

### Lung cellular response

**Figure 2:**
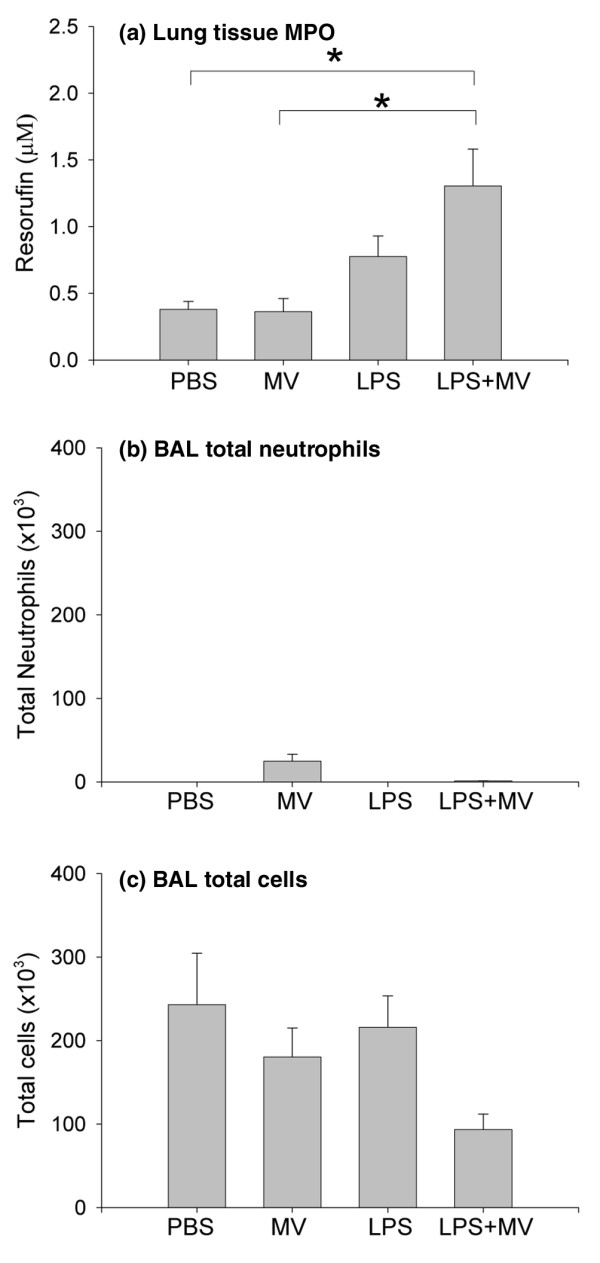
Cellular response. Lung homogenate myeloperoxidase activity **(a)**, bronchoalveolar lavage fluid (BALF) total neutrophils **(b)**, and BALF total cells **(c) **in mice treated with intraperitoneal PBS followed 14 hours later by 6 hours of spontaneous breathing (PBS) or mechanical ventilation (MV), and in mice treated with intraperitoneal lipopolysaccharide (LPS; 1 μg/kg) followed 14 hours later by either spontaneous breathing (LPS) or 6 hours of mechanical ventilation (LPS + MV). In all groups receiving mechanical ventilation, the tidal volume was 10 ml/kg and the fraction of inspired oxygen was 0.21. *p < 0.05.

The lung MPO activity, which measures intravascular and extravascular polymorphonuclear cells, was significantly elevated in the combination group (LPS + MV) than in the mice in the PBS and MV groups (*p *< 0.05; Figure 2a). In contrast, the BALF from animals in all groups contained very few neutrophils (Figure 2b), suggesting that the increase in total lung neutrophils was limited to the vessels and interstitium and was not followed by migration into the airspaces during the six hour experimental period. There was no significant difference between groups in the total number of BALF cells, although there was a trend toward fewer total cells in the LPS + MV group (Figure 2c). Most of the cells in the BALF were alveolar macrophages, regardless of treatment.

### Lung cytokine response

**Figure 3:**
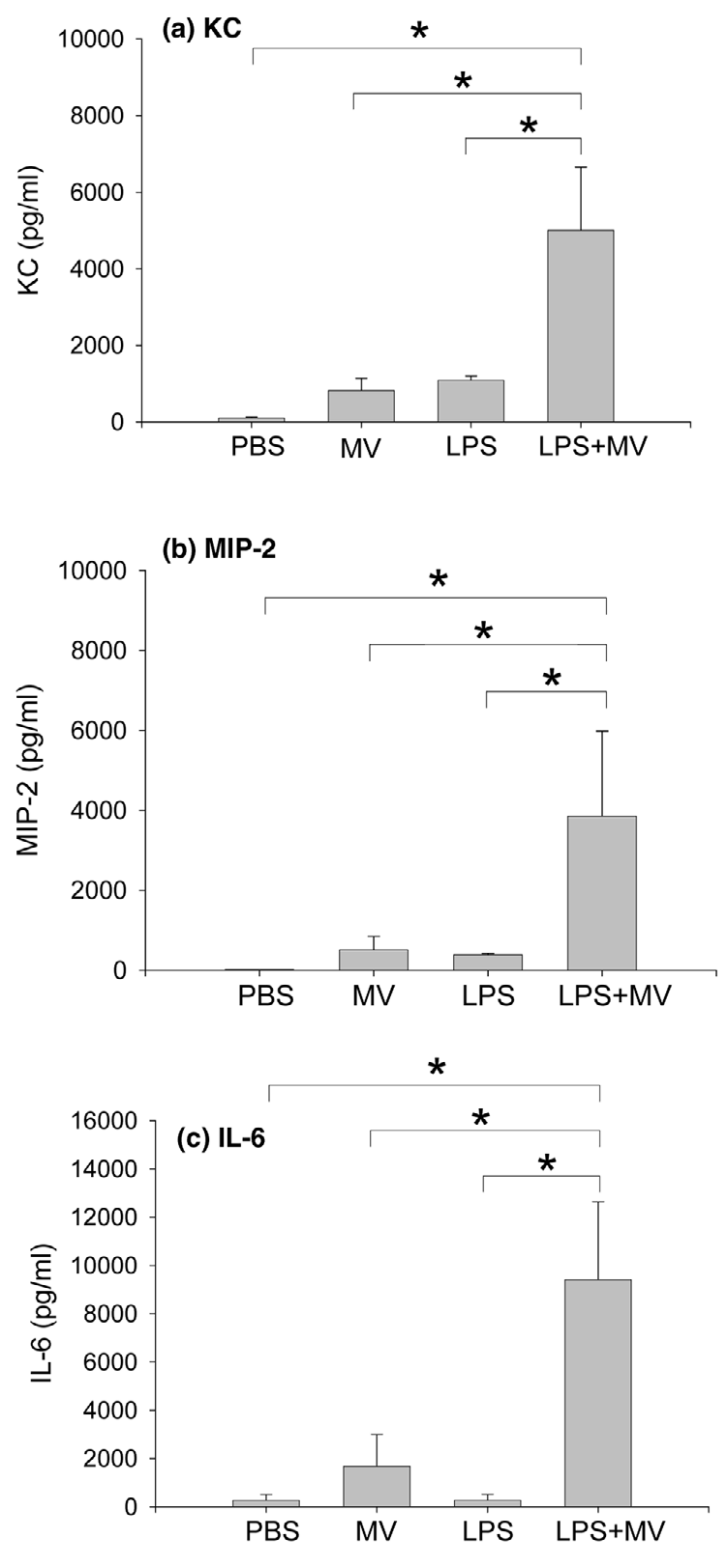
Lung cytokine response. Lung homogenate concentrations of KC **(a)**, macrophage inflammatory protein-2 (MIP-2) **(b)**, and IL-6 **(c) **in mice treated with intraperitoneal PBS followed 14 hours later by 6 hours of spontaneous breathing (PBS) or mechanical ventilation (MV), and in mice treated with intraperitoneal lipopolysaccharide (LPS; 1 μg/kg) followed 14 hours later by either spontaneous breathing (LPS) or 6 hours of mechanical ventilation (LPS + MV). In all groups receiving mechanical ventilation, the tidal volume was 10 ml/kg and the fraction of inspired oxygen was 0.21. *p < 0.05.

Lung homogenates from animals in the combination group (LPS + MV) contained significantly increased concentrations of KC, MIP-2, and IL-6 in comparison with animals in the PBS, MV or LPS groups (Figure 3). IL-1β was detectable in lung homogenates from all groups at similar concentrations (PBS, 210 ± 15 pg/ml; MV, 234 ± 8.6 pg/ml; LPS, 261 ± 15.5 pg/ml; LPS + MV, 349 ± 79 pg/ml). TNF-α was not detected in the lung homogenates of the animals from any of the groups.

### Lung permeability response

Assessment of the integrity of the alveole–capillary barrier was performed by measuring the concentrations of total protein and IgM in BALF (Table 1). The concentrations of IgM in BALF were significantly higher in the MV and the LPS + MV groups than in the PBS group.

### Lung histology

**Figure 4:**
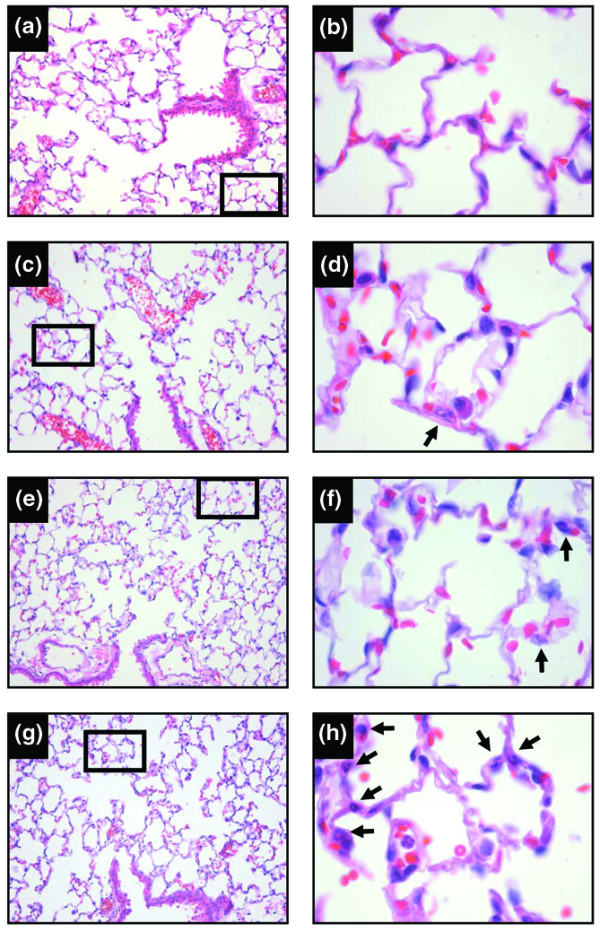
Tissue response. Representative lung tissue sections stained with hematoxylin and eosin, from mice treated with intraperitoneal PBS followed 14 hours later by 6 hours of spontaneous breathing (PBS) **(a, b) **or mechanical ventilation (MV) **(c, d)**, and from mice treated with intraperitoneal lipopolysaccharide (LPS; 1 μg/kg) followed 14 hours later by either spontaneous breathing (LPS) **(e, f) **or 6 hours of mechanical ventilation (LPS + MV) **(g, h)**. The arrows show neutrophil in the alveolar walls. Note the slight thickening of the alveolar walls in (h). The right column shows magnifications of the indicated areas in the left column. Magnifications: left column, ×200; right column, ×400. In all groups receiving mechanical ventilation, the tidal volume was 10 ml/kg and the fraction of inspired oxygen was 0.21.

Histopathological examination of the lungs confirmed an increase in alveolar wall neutrophils in the combination LPS + MV group (arrows), but very few of the polymorphonuclear cells migrated into the airspaces (Figure 4). There was no evidence of intra-alveolar protein deposition in the lungs. Occasional interstitial neutrophils were seen in the lungs from mice in the LPS and PBS + MV groups. Lung architecture was normal in the lungs of mice in the PBS group.

### Lung apoptotic response

Apoptotic activity was measured with immunoblots for cleaved caspase-3 in whole lung homogenates, and also with immunohistochemistry for cleaved caspase-3. There was no evidence of caspase-3 cleavage in the lungs in any of the groups. Soluble FasL was undetectable in the BALF, as measured by immunoassay.

### Systemic cytokine response

**Figure 5:**
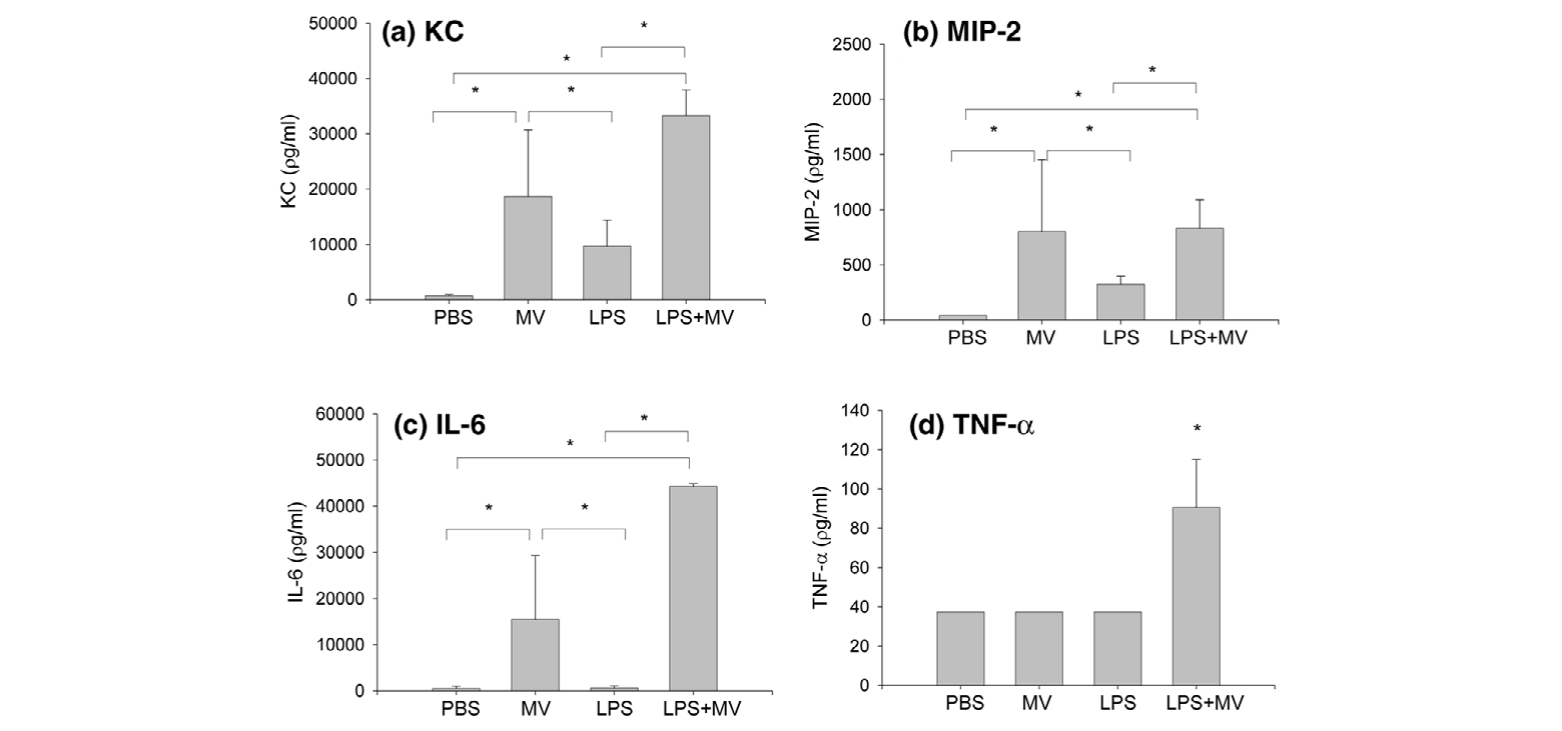
Plasma cytokine response. Plasma concentrations of KC **(a)**, macrophage inflammatory protein-2 (MIP-2) **(b)**, IL-6 **(c)**, and TNF-α **(d) **in mice treated with intraperitoneal PBS followed 14 hours later by 6 hours of spontaneous breathing (PBS) or mechanical ventilation (MV), and in mice treated with intraperitoneal lipopolysaccharide (LPS; 1 μg/kg) followed 14 hours later by either spontaneous breathing (LPS) or 6 hours of mechanical ventilation (LPS + MV). In all groups receiving mechanical ventilation, the tidal volume was 10 ml/kg and the fraction of inspired oxygen was 0.21. *p < 0.05.

The plasma concentrations of KC, MIP-2, and IL-6 were significantly elevated in the combination group (LPS + MV), in comparison with either the PBS group or the LPS group (*p *< 0.05; Figure 5a–d). Interestingly, mechanical ventilation alone (MV) resulted in a significant increase in plasma KC, MIP-2, and IL-6, but not TNF-α, in comparison with the PBS and LPS groups.

### Markers of renal and hepatic function

**Figure 6:**
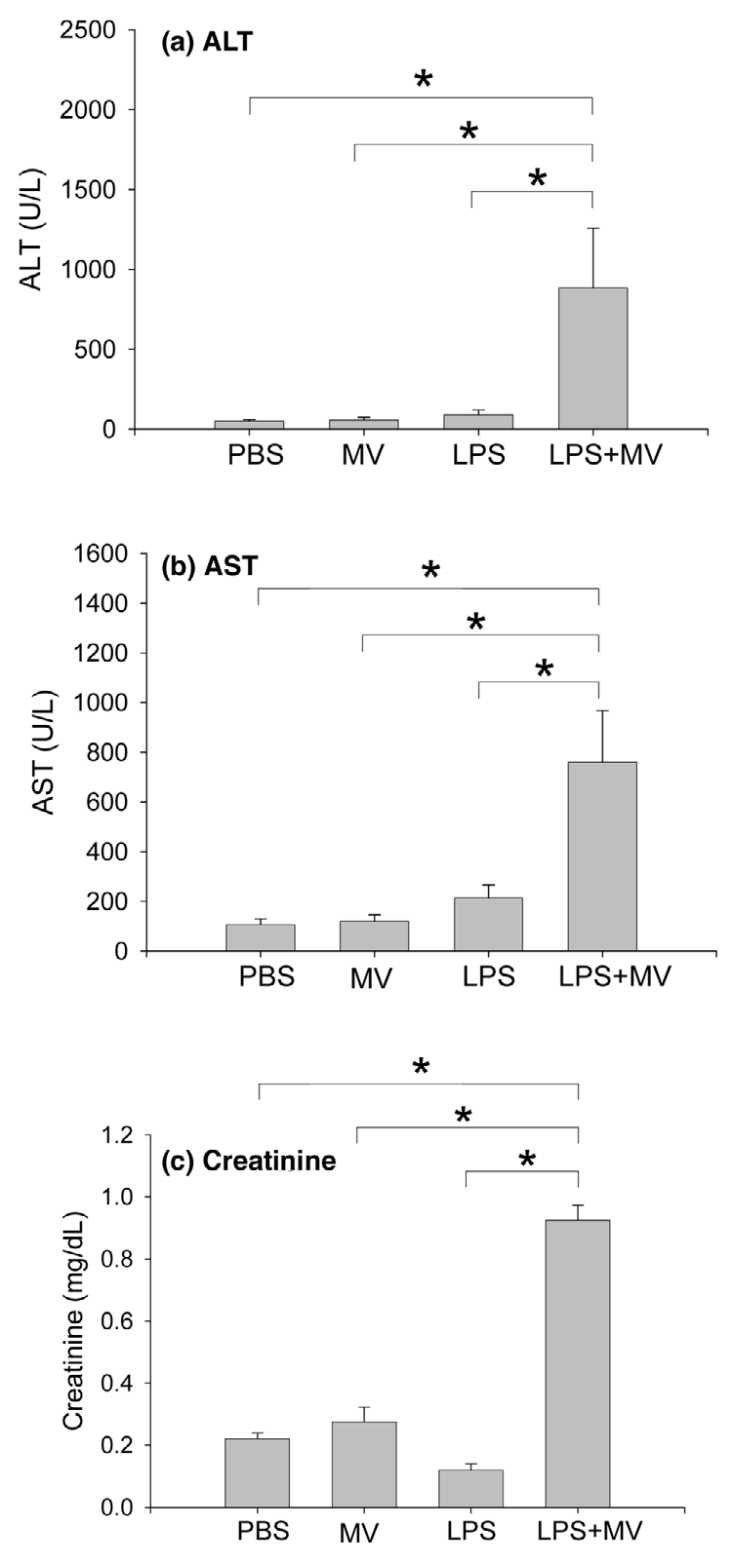
Markers of organ dysfunction. Plasma concentrations of alanine aminotransferase (ALT) **(a)**, aspartate aminotransferase (AST) **(b)**, and creatinine **(c) **in mice treated with intraperitoneal PBS followed 14 hours later by 6 hours of spontaneous breathing (PBS) or mechanical ventilation (MV), and in mice treated with intraperitoneal lipopolysaccharide (LPS; 1 μg/kg) followed 14 hours later by either spontaneous breathing (LPS) or 6 hours of mechanical ventilation (LPS + MV). In all groups receiving mechanical ventilation, the tidal volume was 10 ml/kg and the fraction of inspired oxygen was 0.21. *p < 0.05.

Despite the increase in the plasma cytokine concentrations observed in both ventilated groups, only the animals in the combination LPS + MV group had significant increases in plasma concentrations of the liver injury markers ALT and AST in comparison with each of the other groups (*p *< 0.05; Figure 6a, b). The gamma-glutamyl transpeptidase (GGT) was undetectable in the PBS and LPS groups, and was 5.8 ± 0.4 U/l in the MV group and 15.3 ± 5.3 U/l in the LPS + MV group. Plasma creatinine, a marker of renal function, was significantly increased in the animals in the combination LPS + MV group in comparison with each of the other three groups (*p *< 0.05; Figure 6c).

### Liver and renal histology

**Figure 7:**
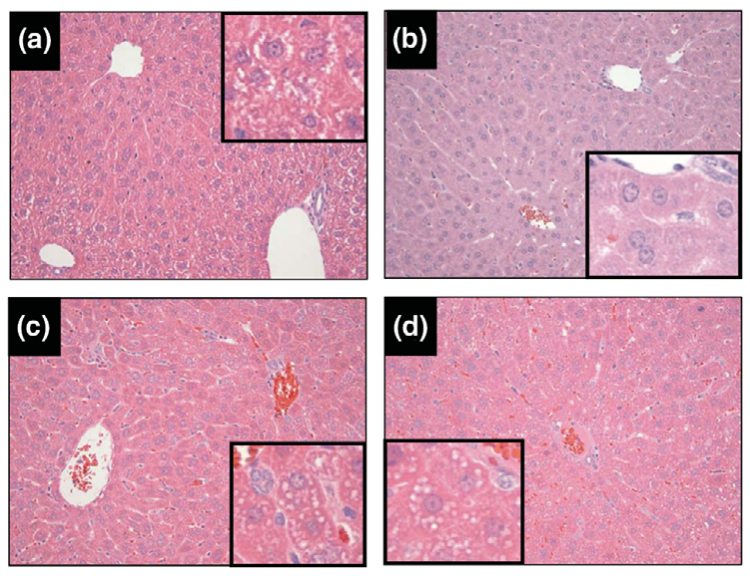
Liver histopathology. Representative samples from liver tissue sections stained with H&E, from mice treated with intraperitoneal PBS followed 14 hours later by 6 hours of spontaneous breathing **(a) **or mechanical ventilation **(b)**, and from mice treated with intraperitoneal lipopolysaccharide (LPS; 1 μg/kg) followed 14 hours later by either spontaneous breathing **(c) **or 6 hours of mechanical ventilation **(d)**. Livers from mice in the PBS group showed normal liver architecture, and cytoplasmic accumulation of glycogen (a, inset). Livers from mice in the mechanical ventilation (MV) group also showed normal architecture and glycogen depletion (b). Mice from the LPS group had accumulation of microvesicles in the cytoplasm (inset), which predominated in the periportal area (c). This microvesicular injury (inset) was also present in the LPS + MV group, but was markedly more extensive (d).

Histological examination of the liver revealed evidence of very mild, scattered microvesicular degeneration, consistent with microvesicular steatosis in the periportal areas of the LPS group (Figure 7c). In the LPS + MV group, the microvesicular steatosis was markedly more severe and diffuse, extending from the portal triads to the central venule (Figure 6d). No other histological lesions were present in this group. There was no evidence of liver injury in the PBS or MV groups (Figure 7a, b). Livers from mice in the PBS group showed intracytoplasmic glycogen deposition (Figure 7a) consistent with liver from non-fasted mice. The kidneys from the mice in the LPS + MV group showed accumulation of protein in the collecting tubules, without evidence of acute tubular necrosis. The kidneys of the animals in the MV and LPS groups were normal.

### Systemic apoptotic response

There was no evidence of apoptosis in the kidneys or livers, as determined by immunohistochemistry for cleaved caspase-3. FasL was below the limits of the assay in plasma from mice in any of the treatment groups.

## Discussion

The goal of this study was to determine whether the combination of mechanical ventilation and low-dose systemic endotoxemia induces the development of distal organ injury. The main finding was that the combination of systemic LPS and mechanical ventilation with tidal volumes of 10 ml/kg resulted in kidney and liver injury, even in the absence of major lung injury. This was associated with increases in the concentrations of plasma cytokines, but not with evidence of apoptosis either in the lungs or in distal organs.

The relationship between mechanical ventilation and distal lung injury was addressed in an important study by Imai and colleagues [7], who noticed that the addition of mechanical ventilation was associated with the development of distal organ damage in rabbits with lung injury. However, that study did not investigate whether mechanical ventilation could enhance the development of distal organ failure in the absence of acute lung injury. This is a clinically relevant issue because many patients with sepsis in the intensive care unit are also ventilated but have not met criteria for ALI or ARDS. In this study we developed a model of ventilated, endotoxemic mice in which neither the ventilatory strategy alone nor the LPS treatment alone resulted in injury of the lungs or distal organs. The combination treatment resulted in molecular and histological evidence of mild injury, as demonstrated by increased lung cytokines and neutrophil entrapment within the lung interstitium, but did not lead to protein deposition or cellular infiltrates within the alveolar spaces. Thus, we believe that our murine model approaches the scenario of the ventilated patient with circulating endotoxin but without the development of lung infiltrates and thus without clinical ARDS. The key finding of the present study is that mice in the combination group (LPS + MV) developed liver and kidney histological damage and increased plasma creatinine, ALT and AST, indicating distal organ dysfunction, in the absence of overt lung injury.

An important caveat of our study is that we were unable to measure systemic blood pressure in our mice. Thus, hypotension or depressed cardiac function may have resulted in decreased organ perfusion, leading to end organ injury. However, the end-organ damage observed was not characteristic of hypoperfusion. Microvesicular steatosis is a rapidly developing change associated with primary or secondary mitochondrial dysfunction and is due to impairment of fatty acid β-oxidation [19-21]. Mitochondrial injury and resulting microvesicular steatosis can be induced by a variety of insults, including various drugs, toxins (including LPS), hormones and metabolic conditions alone or in combination [20-24]. In this study very mild microvesicular injury was present in livers of mice treated with LPS only, whereas livers from mice treated with ventilation alone were normal. When treatment with LPS was combined with ventilation, the microvesicular change was marked. Thus, the evidence suggests that distal organ injury was a direct result of a synergistic combination of mechanical ventilation-induced effects and circulating LPS and was probably not due to hypotension.

Another important finding of our study is that the damage to distal organs was not associated with the development of apoptosis, as seen previously in the study of Imai and colleagues [7]. Those authors used a rabbit model of acid aspiration and mechanical ventilation, and studied the animals after 8 hours. In contrast, we used mice, intraperitoneal LPS, and studied the animals after six hours. The difference in species, model used, and time of study may account for the different observations regarding distal organ apoptosis.

An interesting finding was that the cytokine patterns in the lung and plasma were markedly different: in the lung tissues we observed an increase in cytokine concentrations only in response to LPS + MV, whereas in plasma there was an increase in response to MV alone, in addition to LPS + MV. Furthermore, in the LPS + MV group the lung concentrations of KC and MIP-2 were relatively similar, but KC was much higher than MIP-2 in plasma. These differences in the lung and plasma cytokine patterns suggest that in this model the cytokines were either locally produced or selectively transported, rather than passively moving from one compartment to the other through disrupted barriers.

We propose that circulating neutrophils and monocytes are primed by LPS in the systemic circulation and are further activated by mechanical ventilation (stretch) in the pulmonary circulation, leading to a systemic inflammatory response and development of organ injury. This is followed by the local production of pro-inflammatory cytokines and enhancement of the local inflammatory response (as demonstrated in our model by the cytokine responses in lung tissue). This interpretation is supported by previous observations suggesting that stretch and LPS activate pro-inflammatory pathways through separate but complementary mechanisms [10].

A prevailing paradigm suggests that MODS results from dysregulation of the innate immune response. This view is supported by the finding that higher concentrations of circulating cytokines are associated both with the development of MODS and with increased mortality in patients with MODS [25,26]. Mediators of apoptosis, in particular the Fas/FasL system, have been also associated with the onset of MODS and mortality in humans [27,28]. The present study suggests that mechanical ventilation may enhance the systemic inflammatory response to low levels of circulating endotoxin, even at tidal volumes that do not result in overt lung injury.

## Conclusion

We have developed a murine model of mechanical ventilation in endotoxemic animals, in which neither mechanical ventilation by itself, nor LPS by itself, results in lung or end-organ injury, but in which the combination of LPS with mechanical ventilation results in end-organ injury. A key finding is that distal organ injury occurred in the absence of overt lung injury. This model is clinically relevant because it reproduces the patient who has sepsis and is being ventilated but who has not yet developed ARDS. In our model, the mechanism of distal organ injury was not associated with apoptosis (either in the lungs or distally) but instead with a systemic inflammatory response. We conclude that mechanical ventilation enhances the systemic inflammatory response to low-dose endotoxemia, leading to extrapulmonary end-organ injury.

## Key messages

• Clinically relevant mechanical ventilation enhances the systemic inflammatory response to low dose endotoxemia, leading to extrapulmonary end-organ injury.

## Abbreviations

ALT = alanine aminotransferase; ARDS = acute respiratory distress syndrome; AST = aspartate aminotransferase; BALF = bronchoalveolar lavage fluid; FasL = Fas ligand; IL = interleukin; LPS = lipopolysaccharide; MECO_2 _= mixed expired CO_2_; MIP-2 = macrophage inflammatory protein-2; MODS = multiple organ dysfunction syndrome; MPO = myeloperoxidase; MV = mechanical ventilation; TNF = tumor necrosis factor.

## Competing interests

The authors declare that they have no competing interests.

## Authors' contributions

DSO'M performed the experiments and drafted the manuscript. WCL participated in the design and coordination of the project, assisted with the interpretation of the data, and helped to draft the manuscript. WA participated in the development of the mouse model of mechanical ventilation, participated in the conception of the project, and participated in the analysis and interpretation of the data. CWF assisted with the preparation of the tissue sections and tissue staining. DL participated in the interpretation of the liver and kidney tissue sections. TRM participated in the design and coordination of the experiments. GMB participated in the conception of the study, in the development of the murine models, in the interpretation of the data and in the drafting of the final manuscript. All authors read and approved the final manuscript.

## Figures and Tables

**Table 1:** Concentrations of total protein and IgM in bronchoalveolar lavage fluid

Group	Total protein (μg/ml)	IgM (ng/ml)
PBS (*n *= 5)	113 ± 26	2^a^
MV (*n *= 6)	194 ± 11	43 ± 11^b^
LPS (*n *= 5)	189 ± 12	10 ± 5
LPS + MV (*n *= 4)	190 ± 41	30 ± 20^c^

Results are shown as means ± SEM. MV, mechanical ventilation. ^a^Undetectable. The lower limit of the assay (2 ng/ml) was used for calculations. ^b^*p *< 0.05 compared with the PBS group and with the lipopolysaccharide (LPS) group. ^c^*p *< 0.05 compared with the PBS group.
